# Association between perioperative β-blocker use and clinical outcome of non-cardiac surgery in coronary revascularized patients without severe ventricular dysfunction or heart failure

**DOI:** 10.1371/journal.pone.0201311

**Published:** 2018-08-01

**Authors:** Jungchan Park, Jeayoun Kim, Ji Hye Kwon, Soo Jung Park, Jeong Jin Min, Sangmin Maria Lee, Hyeon-Cheol Gwon, Young Tak Lee, Myungsoo Park, Seung Hwa Lee

**Affiliations:** 1 Department of Anesthesiology and Pain Medicine, Samsung Medical Center, Sungkyunkwan University School of Medicine, Seoul, Korea; 2 Division of Cardiology, Department of Medicine, Heart Vascular Stroke Institute, Samsung Medical Center, Sungkyunkwan University School of Medicine, Seoul, Korea; 3 Department of Thoracic and Cardiovascular Surgery, Samsung Medical Center, Sungkyunkwan University School of Medicine, Seoul, Korea; 4 Department of Internal Medicine, Hallym University Dongtan Sacred Heart Hospital, Hwaseong, Korea; Hospital Universitari Bellvitge, SPAIN

## Abstract

Perioperative use of β-blocker has been encouraged in patients undergoing non-cardiac surgery despite weak evidence, especially in patients without left ventricular systolic dysfunction (LVSD) or heart failure (HF). This study evaluated the effects of perioperative β-blocker on clinical outcomes after non-cardiac surgery among coronary revascularized patients without LVSD or HF. Among a total of 503 patients with a history of coronary revascularization (either by percutaneous coronary intervention or coronary arterial bypass grafts) undergoing non-cardiac surgery, those without severe LVSD defined by ejection fraction over 30% or HF were evaluated. The primary outcome was a composite of death, myocardial infarction, repeat revascularization, and stroke during 1-year follow-up. Perioperative β-blocker was used in 271 (53.9%) patients. During 1-year follow-up, we found no significant difference in primary outcome between the two groups on multivariate analysis (hazard ratio [HR], 1.01; confidence interval [CI] 95%, 0.56–1.82; P = 0.963). The same result was shown in propensity-matched population (HR, 1.25; CI 95%, 0.65–2.38; P = 0.504). In coronary revascularized patients without severe LVSD or HF, perioperative β-blocker use may not be associated with postoperative clinical outcome of non-cardiac surgery. Larger registry data is needed to support this finding.

## Introduction

Coronary heart disease (CHD) is a well-known risk factor of non-cardiac surgery, and nearly half of postoperative mortality is caused by cardiac complications [1,2]. β-blockers, traditionally prescribed for hypertension, are the primary choice of treatment after myocardial infarction (MI) or chronic angina [3,4]. In addition, β-blockers have shown improved clinical outcome in patients with chronic heart failure (HF) [5,6]. In this regard, they have not only been recommended as first line therapy for patients with CHD, but also been proposed to reduce cardiac complications by moderating hemodynamic response in the perioperative period [2,7–9]. However, evidence for these recommendations has been questioned and there remains debate on the cardio-protective effects of perioperative β-blocker in non-cardiac surgery [10]. Recent studies suggest that perioperative β-blocker use may not be associated with postoperative clinical outcome in CHD patients, especially when left ventricular function is preserved [11–13].

We therefore conducted this study to investigate whether perioperative use of β-blocker is associated with postoperative clinical outcome of non-cardiac surgery in coronary revascularized patients without severe left ventricular systolic dysfunction (LVSD) or HF.

## Methods

### Study population and data collection

This study was a single-center retrospective study. From February 2010 to December 2015, 537 patients undergoing non-cardiac surgery were initially enrolled in our registry. Inclusion criteria were: 1) patients who had a history of coronary revascularization either by coronary arterial bypass graft or percutaneous coronary intervention or both, 2) patients who underwent non-cardiac surgery under general anesthesia. Exclusion criteria were 1) patients with HF, and 2) patients with ejection fraction lower than 30%. In patients with multiple non-cardiac surgeries, only the first surgery after coronary revascularization was left for analysis. Preoperative variables, including medications and postoperative clinical outcome data within 1-year follow-up were collected by a trained study coordinator using a standardized case report form. All patients were anonymously analyzed, and the study protocol was approved by the Institutional Review Board of Samsung Medical Center (IRB No. 2017-01-026).

### Definitions and outcomes

During an in-hospital stay, it is institutional policy to investigate all current medications, and convert them to in-hospital prescriptions. After admission, the department of anesthesia independently investigates and double checks current medications, and fills in a standardized preoperative evaluation sheet.

Preoperative evaluation at our institution starts at the outpatient department. Routine poreoperative tests include liver and renal biochemistries, complete blood count, coagulation profile, serologic assays for viruses and plain chest film. Further evaluations including electrocardiography, echocardiography, pulmonary function test, and imaging studies are performed in a multidisciplinary manner if necessary. Along with medications, any past medical history related to risk of surgery is also contained in the preoperative evaluation sheet.

Surgical risk stratification was in accordance with 2014 European Society of Cardiology/Anesthesiology (ESC/ESA) guidelines [2]. Diabetes mellitus was defined as having a history of treatment such as medication and lifestyle intervention or diagnosis of type 1 or type 2 diabetes mellitus. Hypertension was self-reported or prescription of anti-hypertensives or systolic blood pressure >140 mm Hg at rest. Stroke was defined as brain hemorrhage, transient ischemic attack, and cerebral infarction. Dyslipidemia was self-reported or defined as prescription of lipid lowering agents. HF included diastolic heart failure with preserved left ventricular function, and was defined as a history of HF or use of loop diuretics on or during hospital stay.

The primary outcome was major adverse cardiovascular and cerebral events (MACCE), defined as a composite of all-cause death, myocardial infarction, repeat revascularization, and stroke during 1-year follow-up. Repeat revascularization included target vessel and non-target vessel revascularization. The secondary outcomes included intraoperative and postoperative parameters Intraoperative parameters were hypotension, bradycardia, and adrenergic drug requirement. Postoperative parameters were MACCE during 30 days follow-up, and each of MACCE and the composite of all-cause death, myocardial infarction, and repeat revascularization during 1-year and 30-days follow-up.

### Statistical analysis

Continuous variables are presented as mean ± standard deviation (SD) and categorical variables are presented as percentages. Differences between each group were compared using t-test or Wilcoxon rank-sum test for continuous variables, and Chi-square or Fisher’s exact test for categorical variables. Survival curves using Kaplan-Meier estimates were constructed and log-rank test was compared.

We hypothesized that the incidence of MACCE during 1-year follow-up is higher in the β-blocker group compared to the no β-blocker group. Based on previous studies, we expected 10% in the β-blocker group to be reduced to 6% in the no β-blocker group [14]. With 59 months for accrual period and an additional 12 months of follow-up, the power of this study (232 patients in the no beta-blocker group, 271 patients in the beta-blocker group) is about 81% assuming the time to MACCE occurrence is exponentially distributed [15].

Adjusted hazard ratio (HR) with 95% confidence interval (CI) was reported, using Cox regression based on covariates with clinical relevance or P < 0.1. The following were covariates for adjustment: age, sex, hypertension, current smoking, chronic kidney disease, dyslipidemia, and ESC/ESA surgical risk. We performed rigorous adjustments using propensity scores to reduce selection bias and potential confounding factors. After propensity score matching, an absolute standardized difference < 10% for the measured covariate suggested an appropriate balance between the groups. In the propensity score-matched population, we compared HR for outcomes using a stratified Cox regression model. We also performed a subgroup analysis to reveal hidden interaction with history of myocardial infarction, surgical risk, preserved left ventricular systolic function, revascularization strategy, multivessel disease, and chronic kidney disease. Statistical analyses were performed with SAS 9.4 (SAS Institute Inc., Cary, NC, USA) and R 3.0.3 (Vienna, Austria; http://www.R-project.org/). All tests were 2-tailed, and P < 0.05 was considered statistically significant.

## Result

A total of 537 patients were initially enrolled. Twenty-two patients with ejection fraction under 30% and 12 patients with heart failure were excluded. As a result, 503 patients remained for analysis. Types of surgery are summarized in S1 Table.

### Baseline characteristics

Patients were divided into two groups: 271 (53.9%) in the β-blocker group and 232 (46.1%) in the no β-blocker group. Preoperative variables are summarized in Table 1. The incidence of hypertension, current smoking, and dyslipidemia was higher in the β-blocker group. After performing propensity score-matching, matched data set of 197 pairs was generated by 1:1 individual matching without replacement. There was no significant imbalance between the two groups in the propensity-matched population (Table 1).

**Table 1:** Baseline characteristics.

	Entire population				Propensity-matched population		
	β-blocker (N = 271)	No β-blocker (N = 232)	P-value	SMD	β-blocker (N = 197)	No β-blocker (N = 197)	SMD
Male	201 (74.2)	182 (78.5)	0.262	10	149 (75.6)	154 (78.2)	-5.8
Age	68.9 (±8.8)	69.9 (±9.2)	0.222	-10.9	69.4 (±8.5)	69.7 (±9.0)	-2.9
Diabetes	151 (55.7)	135 (58.2)	0.577	-5.3	113 (57.4)	116 (58.9)	-3.1
Hypertension	226 (83.4)	174 (75)	0.02	22.3	157 (79.7)	160 (81.2)	-4.1
Current smoking	25 (9.2)	10 (4.3)	0.031	17	12 (6.1)	10 (5.1)	3.5
Chronic kidney disease	71 (26.2)	45 (19.4)	0.071	15.6	42 (21.3)	44 (22.3)	-2.3
Dyslipidemia	224 (82.7)	174 (75)	0.035	20	156 (79.2)	151 (76.7)	6.7
Valvular heart disease	20 (7.4)	16 (6.9)	0.834	2	15 (7.6)	13 (6.6)	3.9
History of myocardial infarction	68 (25.1)	52 (22.4)	0.482	6.4	41 (20.8)	43 (21.8)	-2.3
History of stroke	46 (17)	37 (16)	0.757	1.9	32 (16.2)	33 (16.8)	-1.4
History of PAD	72 (26.6)	49 (21.1)	0.154	11.7	47 (23.9)	48 (24.4)	-1.2
Multivessel disease	233 (86)	191 (82.3)	0.262	10.3	164 (83.3)	163 (82.7)	1.5
Ejection fraction < 50%	213 (78.6)	190 (81.9)	0.355	-8.2	162 (82.2)	160 (81.2)	2.5
Hemoglobin	12.2 (±2.1)	12.2 (±2.1)	0.926	0.8	12.2 (±2.0)	12.3 (±2.1)	-2.5
Albumin	4.0 (±0.6)	4.5 (±8.8)	0.339	-93.9	4.0 (±0.6)	4.0 (±0.9)	2.2
Medication							
Aspirin	221 (81.6)	178 (76.7)	0.183	13.3	154 (78.2)	154 (78.2)	0
Clopidogrel	140 (51.7)	110 (47.4)	0.342	8.1	98 (49.8)	92 (46.7)	6.1
ACEi/ARB	122 (45)	102 (44)	0.813	1.7	88 (44.7)	85 (43.2)	3.1
ESC/ESA surgical risk			0.055				
Mild	35 (12.9)	48 (20.7)			29 (14.7)	30 (15.2)	
Intermediate	169 (62.4)	127 (54.7)		16.2	120 (60.9)	118 (59.9)	2.1
High	67 (24.7)	57 (24.6)		-0.3	48 (24.4)	49 (24.9)	-1.2
Emergency	35 (12.9)	38 (16.4)	0.272	-10.2	28 (14.2)	29 (14.2)	-1.5
CABG	148 (54.6)	110 (47.4)	0.107	14.1	98 (49.8)	96 (48.7)	2

Data are presented as mean ± standard deviation or n (%). PAD, peripheral arterial disease; ACEi, angiotensin converting enzyme inhibitor; ARB, aldosterone receptor blocker; ESC, European society of cardiology; ESA, European Society of Anaesthesiology; CABG, coronary arterial bypass grafting

### Clinical outcomes

**Fig 1:**
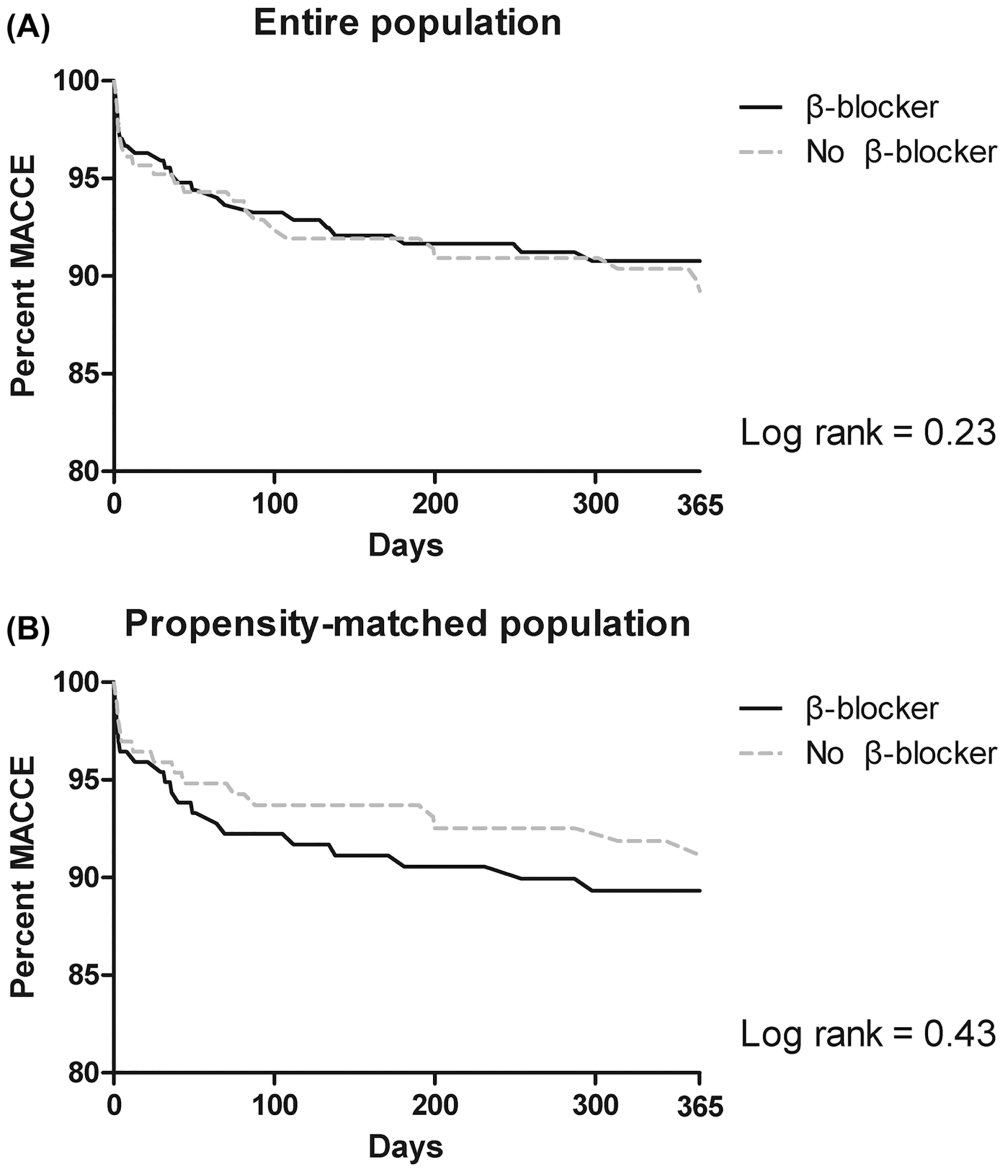
Kaplan-Meier Curves for major adverse cardiovascular and cerebral events during 1-year follow-up in the entire (A) and propensity-matched (B) populations.

On multivariate analysis, the incidence of MACCE on 1-year follow-up was not different between the two groups (HR, 1.01; CI 95%, 0.56–1.82; P = 0.963). The clinical outcomes in entire population are listed in Table 2. The propensity-matched population showed the same results on 1-year follow-up (HR, 1.25; CI, 0.65–2.38; P = 0.34). The results of the analysis in the propensity-matched population are summarized in Table 3. Clinical outcome during 30-days follow-up also showed no significant difference both in the entire and propensity-matched populations. Intraoperative events such as hypotension, bradycardia, and adrenergic drug requirement also showed no significant difference. The incidence of intraoperative events is summarized in S2 Table. Kaplan-Meier Curves for the primary outcome in the entire and propensity-matched populations are shown in Fig 1.

**Table 2:** Clinical outcomes in entire population.

	Univariate analysis				Multivariate analysis	
	β-blocker (N = 271)	No β-blocker (N = 232)	Unadjusted HR (95% CI)	P-value	*Adjusted HR (95% CI)	P-value
1-year follow-up						
MACCE	24 (8.9)	23 (9.9)	0.87 (0.49–1.54)	0.633	1.01 (0.56–1.82)	0.963
All-cause death	12 (4.4)	11 (4.7)	0.90 (0.40–2.05)	0.81	1.26 (0.54–2.95)	0.594
MI	9 (3.3)	5 (2.2)	1.53 (0.51–4.56)	0.448	1.64 (0.54–5.00)	0.385
Stroke	2 (0.7)	2 (0.9)	0.33 (0.06–1.70)	0.185	0.33 (0.06–1.75)	0.193
RR	9 (3.3)	7 (3.7)	1.08 (0.40–2.89)	0.886	1.06 (0.39–2.89)	0.909
Death, MI or RR	22 (8.1)	19 (8.2)	0.97 (0.53–1.80)	0.926	1.16 (0.62–2.18)	0.646
30-day follow-up						
MACCE	11 (4.1)	11 (4.7)	0.85 (0.37–1.97)	0.71	0.95 (0.41–2.24)	0.91
All-cause death	1 (0.4)	7 (3.0)	0.12 (0.02–0.98)	0.048	0.17 (0.02–1.44)	0.105
MI	7 (2.6)	2 (0.9)	3.01 (0.63–14.48)	0.17	3.07 (0.62–15.08)	0.168
Stroke	2 (0.7)	5 (2.2)	0.84 (0.12–5.95)	0.86	0.81 (0.11–5.89)	0.838
RR	8 (3.0)	3 (1.3)	2.28 (0.60–8.58)	0.224	2.21 (0.58–8.45)	0.246
Death, MI or RR	9 (3.3)	10 (4.3)	0.77 (0.31–1.89)	0.567	0.87 (0.35–2.19)	0.773

Data are presented as n (%). MACCE, major adverse cardiovascular and cerebral events; HR, hazard ratio; CI, confidential interval; MI, myocardial infarction; RR, repeat revascularization

*Covariates include age, sex, hypertension, dyslipidemia, chronic kidney disease, current smoking and surgical risk

**Table 3:** Clinical outcomes in the propensity-matched population.

	β-blocker (N = 197)	No β-blocker (N = 197)	Adjusted HR (95% CI)	P-value
1-year follow-up				
MACCE	20 (10.2)	16 (8.1)	1.25(0.65–2.38)	0.504
All-cause death	11 (5.6)	7 (3.6)	1.56(0.62–3.88)	0.343
MI	8 (4.1)	4 (2.0)	2.02(0.60–6.78)	0.255
Stroke	1 (0.51)	3 (1.5)	0.33(0.03–3.18)	0.335
RR	7 (3.6)	7 (3.6)	1.00(0.35–2.89)	0.998
Death, MI or RR	19 (9.6)	14 (7.1)	1.36(0.68–2.72)	0.386
30-day follow-up				
MACCE	9 (4.6)	8 (4.3)	1.13(0.46–2.81)	0.793
All-cause death	1 (0.5)	5 (2.5)	0.20(0.02–1.71)	0.14
MI	6 (3.1)	2 (1.0)	3.03(0.61–15.04)	0.176
Stroke	1 (0.5)	1 (0.5)	0.99(0.06–15.77)	0.994
RR	7 (3.6)	3 (1.5)	2.34(0.60–9.11)	0.22
Death, MI or RR	8 (4.1)	8 (4.1)	1.01(0.40–2.55)	0.991

Data are presented as n (%). MACCE, major adverse cardiovascular and cerebral events; HR, hazard ratio; CI, confidential interval; MI, myocardial infarction; RR, repeat revascularization

**Fig 2:**
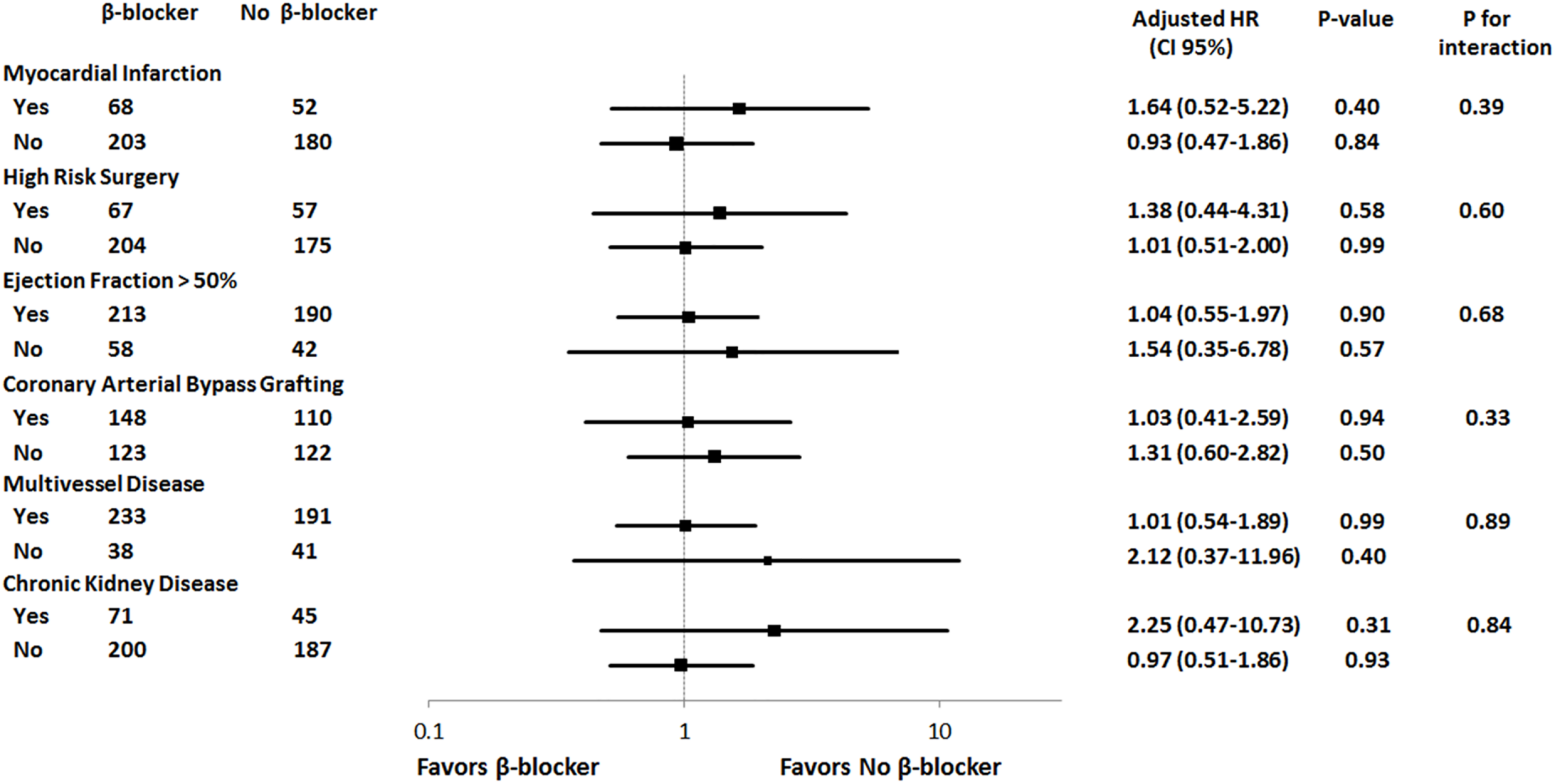
Subgroup analysis of history of myocardial infarction, high-risk surgery, ejection fraction over 50%, coronary arterial bypass grafting as revascularization type, multivessel disease and chronic kidney disease for major adverse cardiovascular and cerebral events on 1-year follow-up.

The result of subgroup analysis is shown in the hazard-ratio plots in Fig 2. There were no significant interactions between perioperative β-blocker use and preoperative variables of interest such as myocardial infarction, surgical risk, preserved left ventricular systolic function, revascularization type, multivessel disease, and chronic kidney disease with respect to the primary outcome.

## Discussion

The present study shows that perioperative β-blocker use may not be associated with postoperative clinical outcomes of non-cardiac surgery in coronary revascularized patients without severe LVSD or HF. This finding suggests that routine use of β-blocker in every coronary revascularized patient during the perioperative period may not be reasonable.

β-blockers reduce key determinants of myocardial oxygen demand—heart rate, blood pressure, and stroke volume. Extrapolation of these benefits is especially valuable during the perioperative period, because surgical stress may cause type II MI. In this respect, current guidelines suggest continued treatment of β-blockers during the perioperative period in patients with previous β-blocker use. The 2002 ACC/AHA guideline was the first to strongly recommend perioperative β-blocker therapy in non-cardiac surgery, and they were mainly based on two randomized clinical trials [16,17]. After few modifications with conflicting evidence, the final recommendation of the 2009 ACC/AHA update was that it is reasonable to initiate β-blocker therapy before intermediate or high-risk surgery only in patients with established CHD [18–20]. The DECREASE (Dutch Echocardiographic Cardiac Risk Evaluation Applying Stress Echocardiography) study has been the main evidence for mortality benefit [8]. However, with questions on the reliability of this study, the efficacy and safety of perioperative β-blocker therapy are still controversial [10]. Recent meta-analyses showed that perioperative β-blocker therapy may not be associated with improved clinical outcome [10,13]. The result of this study corresponds well with these recent results.

The use of β-blocker, as the cornerstone of pharmacotherapy in patients with CHD, is another issue. Other than reducing myocardial oxygen demand, β-blocker increases coronary blood flow with prolongation of diastole, and reduces the burden of angina in patients with obstructive coronary lesion [6,21]. International guidelines recommend use of β-blockers in all patients with acute MI regardless of LVSD or HF [22,23]. However, unlike patients with unstable symptoms in which β-blocker effects are well established, β-blocker effects in stable CHD has been questioned and these guidelines face an enormous challenge, especially in terms of long-term therapy in patients without LVSD or HF [24]. Indeed, most of the evidence of these guidelines predates the era of coronary revascularization. Coronary intervention may make a difference because it potentially preserves more viable and less arrhythmogenic myocardium compared to non-invasive treatment. Moreover, the beneficial effects of β-blocker given early after acute MI in hemodynamically stable patients, is largely driven by a reduction in ventricular arrhythmias and re-infarction, and the mortality advantage after 1 month of use in acute MI patients without LVSD is not known [25]. A meta-analysis on 10 observational studies among more than 40,000 patients showed that the beneficial effects of β-blocker after percutaneous coronary intervention is limited to those with reduced ejection fraction [26]. The most recent study also showed that among survivors after hospitalization due to acute MI without LVSD or HF, β-blocker therapy was not associated with lower all-cause mortality within 1-year follow-up [12].

β-blockers are not completely free from potential harms during the perioperative period. Many patients actually report side effects and many medications show decreased drug adherence [27]. In patients with lowest cardiac risk, perioperative β-blocker use was not associated with any benefit but even with harm [28]. It is also reported that perioperative β-blockers started within one day or less before non-cardiac surgery may be associated with increased risk of bradycardia, hypotension, stroke and even death [1]. We acknowledge that the circumstances of patients in this study are different, in that they pose a certain cardiac risk and showed no side effects of β-blocker from previous use. None of these intraoperative events were increased in this study. However, avoidance of hypotension is still critical in the postoperative period, and β-blocker is no longer recommended as a first line therapy of hypertension [29]. Therefore, in coronary revascularized patients, routine use of perioperative β-blockers may be preserved for ptients who are tolerated by previous use, but not in every patient.

The strength of this study is that it is conducted in patients from coronary revascularization era. The results of our study showing neutral effect of perioperative β-blocker among coronary revascularized patients without severe LVSD, correspond with previous studies on patients with stable CHD and share a potential explanation for the inconsistent effect of β-blocker therapy [11,12]. β-blockers also showed neutral effects in patients undergoing coronary arterial bypass grafts [30]. This study has an adequate setting, which can easily translate the results into everyday clinical practice in the coronary revascularization era. Patients with stable symptoms after coronary revascularization, are commonly scheduled for non-cardiac surgery because prolonged life expectancy leads to higher chances of other pathological conditions requiring surgical treatment [31]. Results of this study showing a neutral effect of perioperative β-blocker in both short-term and long-term follow-up suggest that the addition of β-blocker for coronary revascularized patients during the perioperative period might not be necessary.

Although β-blocker use did not improve overall postoperative clinical outcome, perioperative β-blocker use, according to current guidelines, may have selectively affected those with a history of MI undergoing high-risk surgery. Therfore, we also performed a subgroup analysis to further evaluate interactions with cofactors including history of MI, preserved left ventricular systolic function, and high-risk surgery. In subgroup analysis, no significant interaction was found.

The result of this study must be appraised in the light of following limitations. First, as a nature of retrospective studies, confounding factors may have influenced the results. Even with a propensity score-matched analysis to adjust potential confounding factors, unmeasured factors could not be corrected. Duration of time interval between coronary revascularization and start of β-blocker use might have affected the results, because the benefit of β-blocker is known to be maximal in the early post-MI period. Total duration of β-blocker use could also have affected the results. However, accurate durations were not available in all patients and therefore were not analyzed. Second, the effects of β-blocker initiated immediately before surgery could not be evaluated because this study was only in patients who had been on β-blockers therapy. Third, patients on previous β-blocker might have higher risk, and this might not have been fully adjusted even after propensity matching. Despite these limitations, this is the first study to evaluate the effect of perioperative β-blockers in coronary revascularized patients.

## Conclusion

In coronary revascularized patients without severe LVSD or HF, perioperative β-blocker use may not be associated with postoperative clinical outcome. Large registry data or randomized controlled trials are needed to support this finding.

## Supporting information

S1 Table Types of surgery. (DOCX)

S2 Table Intraoperative events. (DOCX)

## Abbreviations

CHD: coronary heart disease
HF: heart failure
LVSD: left ventricular systolic dysfunction
MI: myocardial infarction
