# Supplementary material for: Association between perioperative β-blocker use and clinical outcome of non-cardiac surgery in coronary revascularized patients without severe ventricular dysfunction or heart failure
Source: PLoS One. 2018 Aug 1;13(8):e0201311. doi: 10.1371/journal.pone.0201311 (PMC6070245; doi:10.1371/journal.pone.0201311)
Supplement: S1 Table — (DOCX) [file pone.0201311.s001.docx]

**S1 Table. Types of surgery**

| **Types of Surgery** | **Number (%)** |
| --- | --- |
| Vascular surgeries | 155 (30.8) |
| Abdominal surgeries | 154 (30.6) |
| Orthopedic surgeries | 69 (13.7) |
| Non-cardiac thoracic surgeries | 53 (10.5) |
| Neurologic surgeries | 23 (4.6) |
| Nasopharyngeal and eye surgeries | 31 (6.2) |
| Urologic and obstetric surgeries | 18 (3.6) |

Data are presented as n (%)
