# Supplementary material for: Association between perioperative β-blocker use and clinical outcome of non-cardiac surgery in coronary revascularized patients without severe ventricular dysfunction or heart failure
Source: PLoS One. 2018 Aug 1;13(8):e0201311. doi: 10.1371/journal.pone.0201311 (PMC6070245; doi:10.1371/journal.pone.0201311)
Supplement: S2 Table — (DOCX) [file pone.0201311.s002.docx]

**S2 Table. Intraoperative Events**

|  | **β-blocker (N=271)** | **No β-blocker (N=232)** | **Unadjusted  HR (95% CI)** | **P-value** |
| --- | --- | --- | --- | --- |
| Hypotension | 35 (12.9) | 34 (14.7) | 0.81 (0.49-1.34) | 0.41 |
| Bradycardia | 133 (49.1) | 99 (42.7) | 1.30 (0.91-1.84) | 0.15 |
| Adrenergic drug requirements | 175 (64.6) | 144 (62.1) | 1.11 (0.77-1.60) | 0.56 |

Data are presented as n (%).
